# Substrate prediction of *Ixodes ricinus* salivary lipocalins differentially expressed during *Borrelia afzelii* infection

**DOI:** 10.1038/srep32372

**Published:** 2016-09-01

**Authors:** James J. Valdés, Alejandro Cabezas-Cruz, Radek Sima, Philip T. Butterill, Daniel Růžek, Patricia A. Nuttall

**Affiliations:** 1 Institute of Parasitology, The Czech Academy of Sciences, Branišovská 31, CZ-37005 České Budějovice, Czech Republic; 2 Department of Virology, Veterinary Research Institute, Hudcova 70, CZ-62100 Brno, Czech Republic; 3 Center for Infection and Immunity of Lille (CIIL), INSERM U1019 – CNRS UMR 8204, Université Lille Nord de France, Institut Pasteur de Lille, Lille, France; 4 Biology Center, The Czech Academy of Sciences, University of South Bohemia, Branišovská 31, CZ-37005 České Budějovice, Czech Republic; 5 Department of Zoology, University of Oxford, Oxford, OX1 3PS, UK

## Abstract

Evolution has provided ticks with an arsenal of bioactive saliva molecules that counteract host defense mechanisms. This salivary pharmacopoeia enables blood-feeding while enabling pathogen transmission. High-throughput sequencing of tick salivary glands has thus become a major focus, revealing large expansion within protein encoding gene families. Among these are lipocalins, ubiquitous barrel-shaped proteins that sequester small, typically hydrophobic molecules. This study was initiated by mining the *Ixodes ricinus* salivary gland transcriptome for specific, uncharacterized lipocalins: three were identified. Differential expression of these *I. ricinus* lipocalins during feeding at distinct developmental stages and in response to *Borrelia afzelii* infection suggests a role in transmission of this Lyme disease spirochete. A phylogenetic analysis using 803 sequences places the three *I. ricinus* lipocalins with tick lipocalins that sequester monoamines, leukotrienes and fatty acids. Both structural analysis and biophysical simulations generated robust predictions showing these *I. ricinus* lipocalins have the potential to bind monoamines similar to other tick species previously reported. The multidisciplinary approach employed in this study characterized unique lipocalins that play a role in tick blood-feeding and transmission of the most important tick-borne pathogen in North America and Eurasia.

Lipocalins are a ubiquitous protein family found in all three domains of living organisms^1^,^2^. Due to low sequence similarity (<20%), classifying and characterizing lipocalins is challenging and constitutes an active area of current research^3^,^4^. Different approaches have been applied to lipocalins based on amino acid sequence^1^, exon-intron-structure^5^, protein structure^4^ and recently, machine-learning^3^. Initial studies suggested that phylogenetic clusters consisted of functionally similar lipocalins^6^. However, it was soon realized that lipocalins show striking functional diversification, with evolutionarily close lipocalins possessing dissimilar functions^1^,^5^. The structure of lipocalins from distantly related taxa may also be conserved^4^. Despite the low sequence identity between lipocalin family members, the lipocalin fold is highly conserved among various species^1^. The characteristic eight-stranded antiparallel beta-barrel tertiary structure forms a binding pocket that carries a variety of ligands, often hydrophobic, which contributes to the function of these small extracellular proteins. Many lipocalins are also multifunctional, through protein-protein interactions^7^, and by forming oligomers^8^.

Largely because of the composition of tick saliva and their epidemiological impact as pathogenic vectors^9^, tick salivary glands are a major focus of high-throughput sequencing^10^. The recently published genome of the North American vector of tick-borne pathogens, *Ixodes scapularis*^11^, and several tick salivary transcriptomes^12^ show that lipocalins are a protein family with a large expansion in ticks. Tick lipocalins are considered outliers since they lack the three structural conserved motifs typical of the general lipocalin family^13^. Besides lipocalins, tick saliva contains an arsenal of macromolecules that evade host defense mechanisms to facilitate blood feeding. This evasion indirectly provides a gateway at the host-tick interface for pathogens such as *Borrelia* spp. and tick-borne encephalitis virus. A profile during viral infection showed that nymphal *I. scapularis* salivary gland lipocalin genes are differentially expressed when compared to mock infections^14^.

There are currently over a dozen salivary lipocalins from various arthropod species structurally resolved in the Protein Databank (PDB)^15^. The hard tick *Rhipicephalus appendiculatus* has two salivary lipocalins that are structurally resolved, bind different ligands and have separate functions, namely, Ra-HBP2 and Japanin. The Ra-HBP2 has been proposed for therapeutic use since it sequesters two histamine molecules, with different affinities, thereby reducing inflammatory responses^16^. Japanin reprograms dendritic cells (antigen-presenting cells) so they no longer respond to a wide spectrum of stimuli that are specific pathogenic markers for immune recognition^17^. The resolved structure of Japanin in the PDB is also bound with cholesterol. Ticks use their salivary components to conceal or camouflage their presence from their host, e.g., by reducing inflammation and reprogramming dendritic cell responses. Another example of tick “camouflage” is provided by coversin (or OmCI), a salivary lipocalin from the soft tick, *Ornithodoros moubata*. The OmCI directly inhibits complement (C5) activation thereby circumventing the host innate immunity^7^. Pharmaceutical applications for OmCI are being explored since it prevents autoimmune myasthenia gravis in rats^18^ and also possesses antiinflammatory properties by binding leukotriene B4 (LTB4)^19^. Other soft ticks and the hard tick *Ixodes ricinus* (the European sister tick to *I. scapularis*) also express salivary lipocalins that bind LTB4^20^,^21^,^22^.

At the biophysical level, molecular dynamics simulations have complemented experimental studies by simulating protein and protein-ligand (intra)interactions at millisecond timescales with high resolution^23^. This type of analysis has advanced drug discovery and a deeper understanding of molecular systems. For instance, competition for ligand/drug binding between two proteins (or pharmacological promiscuity) is extensively studied experimentally, but studies are scarce on the all-atom exploration of this competition. Since tick salivary lipocalins sequester many host molecules involved in defense mechanisms^9^, the Protein Energy Landscape Exploration (PELE) algorithm^24^ was used to demonstrate virtually the pharmacological promiscuity of histamine between the tick lipocalin Ra-HBP2 and the human histamine receptor at the host-tick interface^25^. Our study was initiated by mining for specific lipocalins in the *de novo* transcriptome from the most important European tick disease vector, *I. ricinus*^26^. Structual analysis and biophysical simulations of the lipocalins narrowed the scope of putative ligands. Genetic expression of these *I. ricinus* lipocalins was also examined during feeding at distinct life cycle stages and in response to *Borrelia afzelii* infection.

## Results and Discussion

### Identifying three lipocalins from the European tick disease vector, *Ixodes ricinus*

The most frequent transcripts from the *I. ricinus* salivary gland transcriptome shotgun assembly (TSA: GADI00000000.1) are lipocalins (5.76% of the entire transcriptome) and most (~90%) are putative histamine-binding proteins^26^ according to the Conserved Domain Database (CDD)^27^ and Pfam^28^. This designation by CDD and Pfam suggest that these lipocalins possess the histamine-binding protein fold, but not necessarily an affinity for histamine^29^. Instead, Mans *et al*.^29^ proposed a degenerated pattern motif, CD[VIL]X(7,17)EL[WY]X(11,30)C, to search for tick monoamine-binding lipocalins. Using the degenerated motif in a Pattern Hit Initiated (PHI)-BLAST resulted in ~200 sequences for monomine or monotonin in the non-redundant tick TSA database, mainly from *I. ricinus*. How can specific outliers from the histamine-binding protein fold, or the degenerated motif, be identified in the *I. ricinus* salivary gland transcriptome? Will these outliers still have the potential to be monoamine-binding lipocalins?

Preston *et al*.^17^ proposed that Japanin homologs are confined to metastriate ticks and are not found in prostriate species such as *I. ricinus*. As a deviant from the histamine-binding protein fold and the degenerated motif, the Japanin protein sequence was used in a protein BLAST implemented in the VectorBase website (https://www.vectorbase.org/) to search for specific lipocalin outliers in the *I. ricinus* salivary gland transcriptome. Sequence identity ~25% suggests homology and structural similarity^30^, therefore a homology criteria of >50% coverage and >20% identity was used in the protein BLAST search. No Japanin *I. ricinus* homologs were identified (<20% identity). Next, several unpublished, Japanin-like sequences from different metastriate hard tick species were used as BLAST queries. These BLAST searches identified eight *I. ricinus* sequences with ~25% identity to the metastriate Japanin-like sequences. These eight *I. ricinus* sequences were putatively classified as lipocalins in the initial *de novo* transcriptome by Schwarz *et al*.^26^. Four of the eight *I. ricinus* lipocalins possess the histamine-binding protein fold^26^ according to the CDD and PFAM with confident E-values (7E-015 - 3E-008). The remaining four (protein accession #s JAA67230, JAA65433, JAA70156 and JAA67401) had extremely poor E-values (>0.4). None of the eight *I. ricinus* lipocalins were found in the aforementioned PHI-BLAST since they lack the degenerated pattern motif described by Mans *et al*.^29^. Many identified *I. ricinus* lipocalins are also highly glycosylated^31^; however, according to Schwarz *et al*.^26^, only three of the eight have glycosylation sites. These include, JAA70260 (4 sites), JAA70156 (1 site) and JAA67401 (1 site).

**Figure 1:**
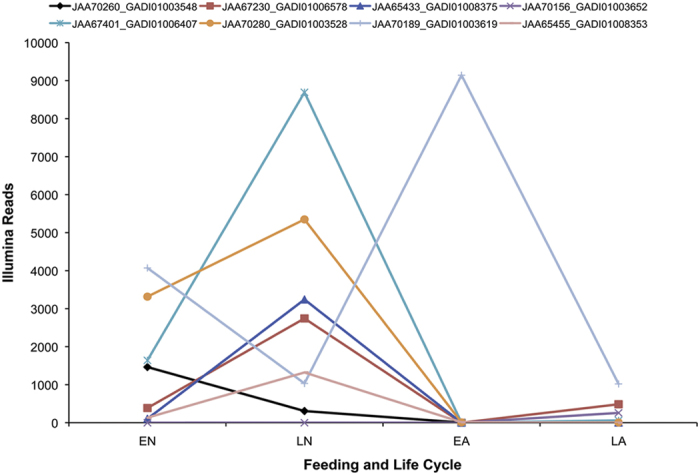
Illumina reads for the *I. ricinus* salivary lipocalins. Data were collected for all eight lipocalin genes from the Illumina reads by Schwarz *et al*.^26^ of the *I. ricinus* salivary gland transcriptome. The y-axis indicates the number of Illumina reads for each color-coded *I. ricinus* lipocalin. The x-axis indicates the feeding and life cycle. EN/EA = early nymph/adult (within 24/48 hours of feeding); LN/LA = late nymph/adult (within 4/7 days of feeding).

The Illumina data from the Schwarz *et al*.^26^ publication was compiled to initially evaluate the expression patterns of the eight identified *I. ricinus* lipocalins. The Illumina reads for the lipocalins in Fig. 1 show the four independent cDNA libraries from the *I. ricinus* salivary gland transcriptome - two nymphal stages (EN and LN) and two adult stages (EA and LA). The specifics of these *I. ricinus* cDNA libraries can be found in Schwarz *et al*.^26^. Except for the JAA70189 gene, the remaining seven lipocalin genes show extremely low Illumina reads during adult blood-feeding (Fig. 1). To further investigate the expression of these genes during adult tick feeding, the tissues of dissected, semi-engorged *I. ricinus* females were examined by quantitative real-time PCR (qPCR). The primer sequences for qPCR are listed in Supplemental Table S1. The qPCR analysis revealed that only three of the eight lipocalin genes (protein accession #s: JAA65433, JAA67401 and JAA70189) were expressed in tissues of the semi-engorged adult female ticks. These three lipocalins were predominantly expressed in the salivary glands, while one gene (JAA65433) was also marginally expressed in tick midgut and ovaries. The discrepancies between the Illumina reads (Fig. 1) and the qPCR results (summarized in Supplemental Table S2) is likely due to the specific tick life cycle and feeding stage analyzed for gene expression. In addition, several studies comparing next-generation sequencing and qPCR results showed some inconsistencies among tick genes^32^,^33^.

### Sequence-based classification of the three *I. ricinus* salivary lipocalins

**Figure 2:**
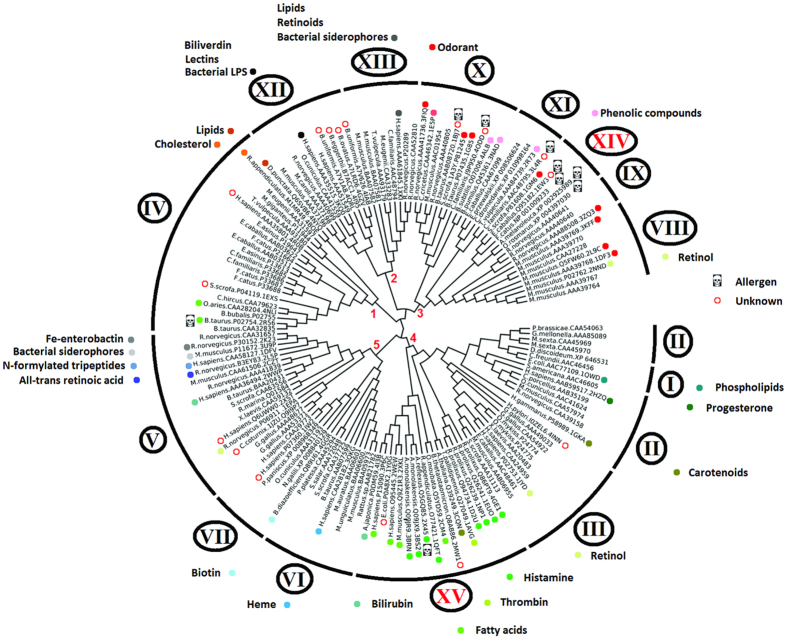
Sequence-based phylogenetic tree of the lipocalin protein family. The ML phylogenetic tree obtained from 143 lipocalins amino acid sequences aligned with PAGAN is shown. The fifteen clusters are marked with Roman numerals (I - XV). Details on Cluster XV are given in Supplemental Fig. S1. The five superclades are also shown in red Arabic numbers (1–5). Known ligands of the lipocalins with available structures in the PDB are shown (colored circles). Lipocalins known to be allergens are also shown (skull symbol). Bootstrap values are available in Supplemental Table S3. Sequences are labeled with species names, GenBank accession numbers and PDB ID (when crystal structures were available).

Lipocalins represent a diverse protein family that has proved difficult to include in a full-family phylogenetic analysis due to high sequence diversity. To obtain a reliable lipocalin alignment (as defined using GUIDANCE), three alignments were produced using the PAGAN, MAFFT, and CLUSTALW algorithms. For a sequence-based phylogeny of lipocalins, the three amino acid sequence alignments (see Materials and Methods) were used to build NJ, ML and MP phylogenetic trees. Based on the bootstrap values of the clusters (Supplemental Table S3), the best topology was produced by the ML using the PAGAN alignment (Fig. 2). The 13 lipocalin clusters proposed by Ganfornina *et al*.^1^ were obtained with bootstraps values higher than 90, except for cluster V that showed lower bootstrap value (Supplemental Table S3). In addition, two new clusters were distinguished, XIV and XV. As shown in Figure 2, 5 superclades were identified that comprise: (1) cluster IV, (2) clusters XII and XIII, (3) clusters VIII - XI and XIV, (4) clusters I - III and XV and (5) VI, VII and X. It is noteworthy that Cluster XIV was consistently recovered in all topologies. Cluster XV, however, was divided into four small subclusters (XVa - XVd; Supplemental Fig. S1) that were consistently recovered using all phylogenetic methods with the PAGAN alignment.

The first study producing a full-lipocalin-family phylogenetic tree^1^ used a gap penalty mask to account for the conserved folding and obtained a reliable tree despite extensive divergence in the primary sequence. Using PAGAN, which is a phylogeny-aware alignment algorithm that is not affected by the inclusion of more diverged sequences in the data set^34^, an alignment was obtained that produced a phylogenetic tree consistent with that of Ganfornina *et al*.^1^. To classify the three salivary lipocalins from *I. ricinus* in the context of the lipocalin clusters, a subsequent PAGAN alignment was performed using all sequences from Fig. 2 together with JAA67401, JAA65433 and JAA70189. The ML phylogenetic tree revealed that the *I. ricinus* sequences JAA67401 and JAA65433 grouped in Cluster IV, together with Japanin, the lipocalin from *R. appendiculatus* that binds cholesterol. The sequence JAA70189, however, did not fall into any of the clusters in Fig. 2 (data not shown).

**Figure 3:**
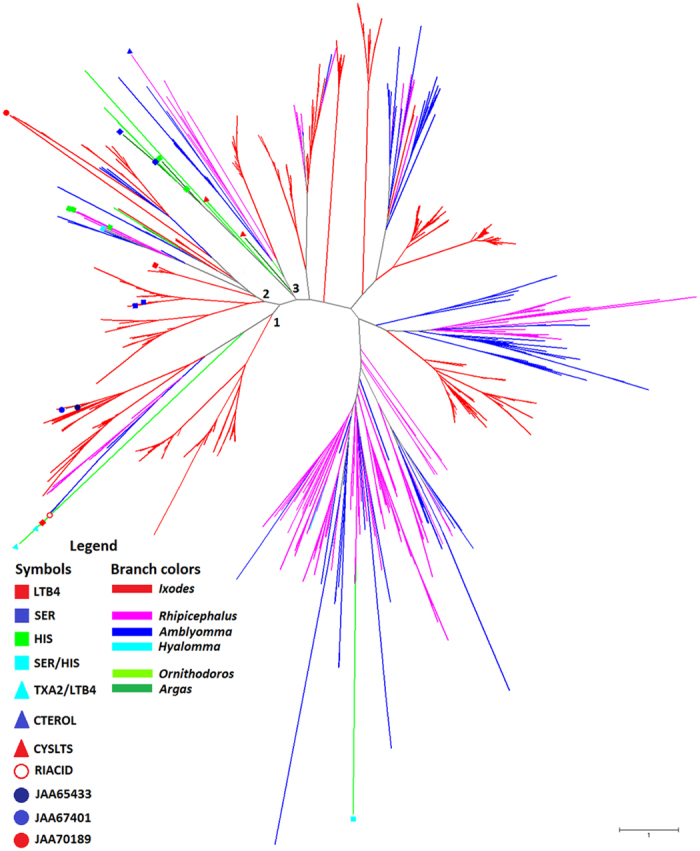
Sequence-based phylogenetic tree of tick lipocalins. The NJ phylogenetic tree obtained from 803 tick lipocalin amino acid sequences aligned with MAFFT is shown. Different tick genera are depicted as colored branches. Tick lipocalins with known ligands (geometric colored symbols) were included in the analysis. Abbreviations for substrate are as follow: ricinoleic acid (RIACID), leukotrienes B4 (LTB4), thromboxane A2 (TXA2), histamine (HIS), serotonin (SER), cholesterol (CTEROL) and cysteinyl leukotrienes (CYSLTS). Clusters 1, 2 and 3 are shown in details in Supplementary Figure S2. The full tree, including sequence accession numbers and bootstrap values, is available in newick format in Supplemental File S1.

To further assess the phylogenetic relation of the three *I. ricinus* lipocalins with other tick lipocalins, we performed a second phylogenetic analysis that included 1748 tick lipocalins available in GenBank. After applying some selection criteria to the lipocalins (see methods), the final alignment contained 803 sequences. The NJ phylogenetic tree obtained from this analysis is shown in Fig. 3. The full phylogenetic tree is available in newick format in Supplemental File S1. As in the previous analysis (Fig. 2), JAA67401 and JAA65433 clustered together, however, they cluster with other lipocalins from *Ixodes*, *Amblyomma*, *Rhipicephalus* and *Ornithodoros* ticks, and not Japanin (Cluster 1, Fig. 3 and Supplemental Figure S2). The lipocalins from *Ornithodoros* in Cluster 1 were previously shown to bind ricinoleic acid (AAT65682 [OmCI]), LTB4 (AAN76829 [TSGP2]) and LTB4 and thromboxane A2 (AAN76830 [TSGP3] and AAA29432 [moubatin]), but not histamine. The *I. ricinus* lipocalin JAA70189 clusters with *Ixodes*, *Amblyomma*, *Rhipicephalus* and *Ornithodoros* lipocalins (Cluster 2, Fig. 3 and Supplemental Figure S2). However, Cluster 2 was enriched with lipocalins that were previously shown to bind histamine (AAC63108 [MS-HBP], AAC63106 [FS-HBP] and AAC63107 [FS-HBP]), serotonin (AAY66602 and AAY66600), histamine and serotonin (AAL56644) and LTB4 (CAJ20733 [Ir-LBP]). Finally, Japanin clustered (Cluster 3, Fig. 3 and Supplemental Figure S2) together with lipocalins that bind cysteinyl leukotrienes (AAN76831 [TSGP4] and ABI52653 [AM-33]), histamine (AJ697694 [Arg r1] and ABI52634 [Monomine]) and serotonin (ABI52694 [Monotonin] and ABI52654).

### Structure-based classification of the three *I. ricinus* salivary lipocalins

**Figure 4:**
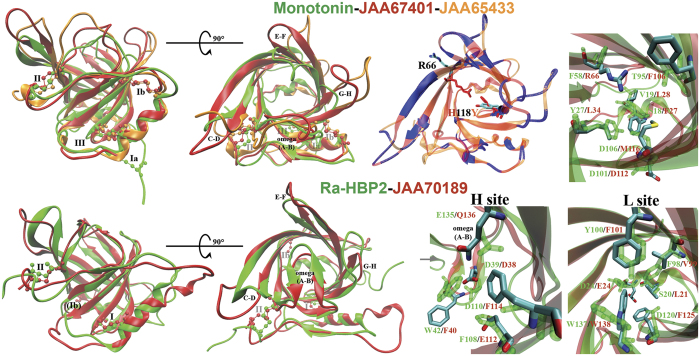
Lipocalin tertiary structures and monoamine binding sites. The *I. ricinus* tertiary models JAA67401 (red) and JAA65433 (orange) are superimposed onto Monotonin (green). Below, the JAA70189 model (red) is superimposed onto Ra-HBP2. The disulfide bridges are represented in roman numerals shown as balls and sticks. The 90° turn is a birds-eye view of the open-end with the beta-hairpin loops labeled. The superimposed JAA67401 (red) and JAA65433 (orange) show the 29% non-conserved residues (blue), the orientation of Arg66 and the substitution His118Tyr. To the right are the specific residues within the binding cavity that form contacts with serotonin (Monotonin) and histamine (Ra-HBP2). The *I. ricinus* lipocalin residues are atom color-coded (carbon = cyan; nitrogen = blue; oxygen = red; sulfur = yellow); hydrogen atoms are not shown.

The Robetta server is one of the top protein structure prediction software tools currently available with high performance in the competition for Critical Assessment of protein Structure Prediction (CASP)^35^. For both JAA65433 and JAA70189 the Robetta server used Ra-HBP2^16^ as a template. The serotonin-binding lipocalin, Monotonin from the soft tick *Argas monolakensis*^29^ was used to model JAA67401. All three *I. ricinus* lipocalins had high confidence values with their respective templates. The homology-modeled structures of the three *I. ricinus* salivary lipocalins are represented in Fig. 4 superimposed onto Monotonin or Ra-HBP2. All three *I. ricinus* lipocalins have an average alpha-carbon backbone root mean square deviation (RMSD) of 2.27 Å ± 0.38 Å with Ra-HBP2 and an average of 2.53 Å ± 0.23 Å RMSD with Monotonin. There is a ~2.3 Å backbone RMSD between the three *I. ricinus* lipocalins. The general structural differences between the three lipocalins are in the beta-hairpin loops of beta-sheets A-B (omega) and G-H of the open-end, and in the disulfide bridges (Fig. 4). Although JAA65433 was modelled after Ra-HBP2 it is structurally similar to Monotonin, as is JAA67401. Compared to Monotonin, however, both JAA67401 and JAA65433 have longer G-H loops and differ in the position of the first disulfide bridge. Additionally, the beta-hairpin loops A-B, E-F and G-H at the open-end of JAA65433 have an outward conformation compared with JAA67401 - the opposite is seen in loop C-D. Compared to Ra-HBP2, JAA70189 has a shorter G-H loop and an unpaired, solvent-exposed, i.e., away from the binding cavity, Cys residue (Ib) near the closed-end (Fig. 4).

The most heterogeneous beta-hairpin loop at the open-end of lipocalins, where ligands usually enter, is the N-terminal omega-loop^16^. The omega-loop of Ra-HBP2 is distinct among all known lipocalins since it is extended and occludes the open-end, suggesting that histamine must enter the ‘barrel’ via some other route^16^. The omega-loop is not extended in the serotonin-binding Monotonin from *A. monolakensis*^29^, JAA67401 or JAA65433, but occludes the open-end of the beta-barrel in JAA70189, similar to Ra-HBP2 (Fig. 4). Another distinction of Ra-HBP2 is that it binds two histamine molecules, one at a higher affinity (H) site near the open-end and another at a lower affinity (L) site near the closed-end^16^. The histamine-binding Monomine from *A. monolakensis* is also lacking the extended omega-loop and only binds one histamine molecule at the L site^29^. The omega-loop of Ra-HBP2 has a few residues that coordinate histamine at the H site^16^. Specific serotonin-binding residues have also been resolved in the Monotonin and Monomine structures^29^. All three *I. ricinus* lipocalins have both structurally conserved positions and substitutions among these monoamine-binding residues of Ra-HBP2 and Monotonin (Fig. 4). The substitutions, however, may prevent the three *I. ricinus* lipocalins maintaining monoamines within their binding cavity or may provide alternative binding modes.

Mans *et al*.^29^ proposed a conserved Asp residue found in Monotonin (Asp101), Monomine (Asp94) and Ra-HBP2 (Asp120) at the L site that may act as a marker for monoamine-binding. This Asp residue is conserved (Asp112) in JAA67401 and JAA65433, but not JAA70189, which has a Phe substitution at position 125 (Fig. 4). On the same JAA70189 beta-hairpin loop as Phe125, however, there is an Asp120 exposed toward the solvent. The superimposed structure in Fig. 4 of JAA67401 (red) and JAA65433 (orange) depict their 71% identity; the remaining 29% (in blue) are generally in the beta-sheets and beta-hairpin loops near the open-end composed of residues oriented towards the solvent. Two structural distinctions within the L site may affect ligand specificity between JAA67401 and JAA65433: (1) the orientation of Arg66 in beta-sheet C is either towards the binding cavity (JAA67401) or towards the solvent (JAA65433), and (2) the substitution in beta-sheet G at position 118 (JAA67401-JAA65433 His118Tyr). The His118Tyr substitution is mirrored from the main structural difference in beta-sheet C of Monomine (Tyr51) and Monotonin (Phe58)^29^, which is substituted by Arg66 in JAA67401/JAA65433. The Arg of Monotonin (Arg109) and Monomine (Arg97) in beta-sheet G are oriented towards the solvent at the structural position His118Tyr in JAA67401/JAA65433. Except for the conserved Asp monoamine-binding marker^29^, the remaining residues of Monotonin that form contacts with serotonin are substituted at the same structural positions in JAA67401 and JAA65433. At the primary sequence level, both the JAA67401 and JAA65433 have the conserved Tyr35 and Thr105 (Tyr27 and Thr95 in Ra-HBP2, respectively) exposed towards the solvent.

The residues of Ra-HBP2 that form contacts with both histamine molecules at the H and L site are more similar and conserved at the structural positions in JAA70189 (lower panel in Fig. 4). At the H site, the omega-loop of JAA70189 has the conserved Asp38 that forms contact with the amine group of histamine in Ra-HBP2 (Asp39) and an aromatic residue (Phe40) that may form pi-pi stacking with the imidazole, as in Trp42 of Ra-HBP2^16^. Two other Ra-HBP2 residues that form pi-pi stacking and contact the amine are Phe108 and Asp110, respectively^16^, the positions of which are swapped in JAA70189 (Glu112 and Phe114). At the L site, JAA70189 has the conserved Trp138 (as the Trp137 pi-pi stacking of Ra-HBP2), the acidic residue (Asp24/Glu24) that forms contacts with the amine and the imidazole ND1, and the aromatic residue (Tyr100/Phe101) with the imidazole ND2 (Fig. 4)^16^. The remaining three histamine-interacting residues at the L site of Ra-HBP2 are not conserved in JAA70189 (Ser20/Leu21, Phe98/Val99 and Asp120/Phe125).

### Ligand screening of the three *I. ricinus* salivary lipocalins

The PELE simulations were initiated as a local exploration (i.e., minimal ligand translation or perturbation) with the respective active site as the ligand starting position (see Materials and Methods). The hypothesis for ligand screening using the Metropolis Monte Carlo biophysical (PELE) simulations was that a specific ligand maintains its relative distance from the active site depending on the lipocalin. Given the local exploration used in the PELE simulations, significantly extended ligand distances indicate that the lipocalin may not sequester that specific ligand. Since the monoamine-binding tick lipocalins Ra-HBP2^16^ and Monotonin^29^ were used as templates for modeling, both structures (and their respective monamines) were included as positive controls in the initial PELE simulations. Since Ra-HBP2 binds two histamine molecules, the starting position for the first set of PELE simulations was at the H site due to its higher affinity. As a negative control for the simulations the Bilin-binding protein (BBP)^36^ from the butterfly *Pieris brassicae* was used since it does not sequester monoamines (i.e., histamine or serotonin). Japanin-cholesterol complex^17^ was also used since the three *I. ricinus* salivary lipocalins were identified using Japanin-like sequences.

**Figure 5:**
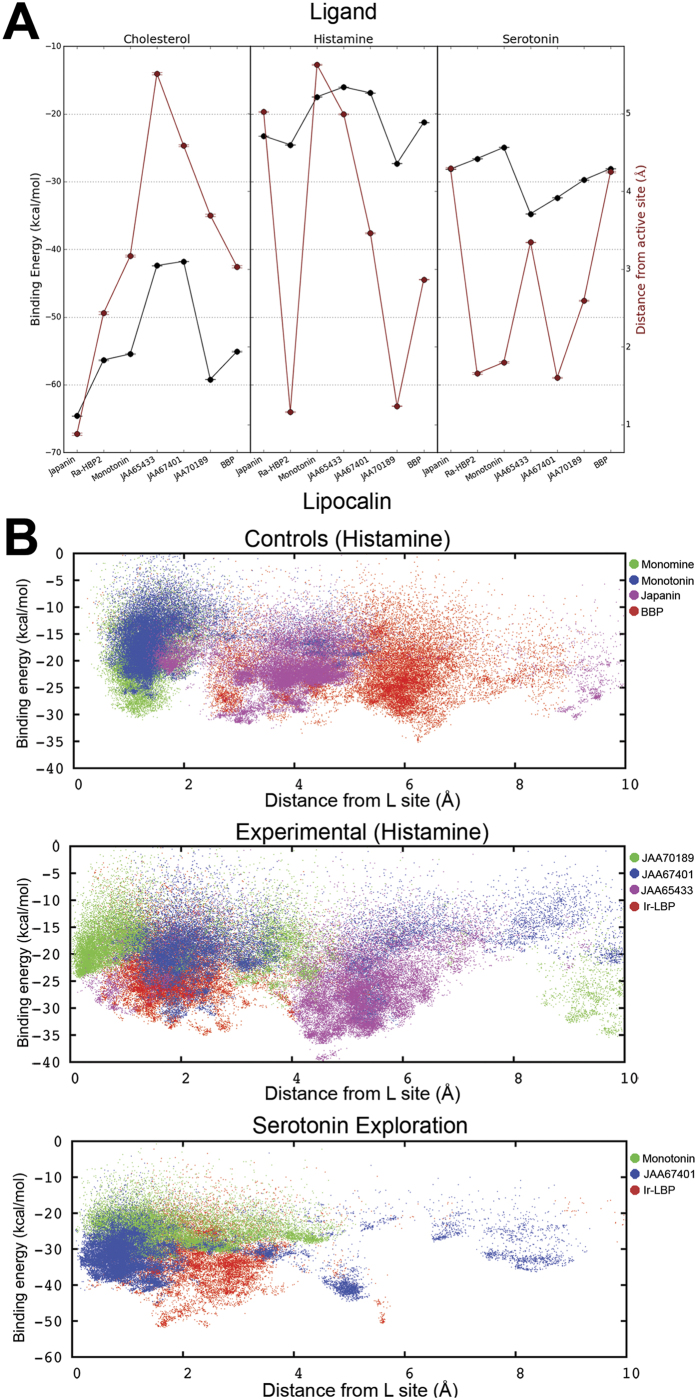
Ligand screening of the three *I. ricinus* lipocalins. Panel A are effect sizes during the biophysical simulations for ligand screening. The two y-axes plot shows the binding energy in kcal/mol (black) and the distance in Å from the respective lipocalin (x-axis) active site (red). The ligands, cholesterol, histamine and serotonin are labeled on top. Panel B are x-y scatter plots of PELE-monoamine explorations at the L site position of Monomine and of Monotonin. The upper plot represents the histamine crystal control systems that include Monomine (green), Monotonin (blue), Japanin (purple) and BBP (red). The middle plot is the histamine explorations of *I. ricinus* lipocalins JAA70189 (green), JAA67401 (blue), JAA65433 (purple) and Ir-LBP (red). The lower plot is the serotonin explorations of Monotonin (green) and the *I. ricinus* lipocalins JAA67401 (blue) and Ir-LBP (red).

Throughout the biophysical simulations for ligand screening, the effects of each lipocalin, the ligand and their interactions on the dependent variable for the binding energy and distance from the active site were highly significant (*P-value* < 0.0001; Table 1). However, with large sample sizes (>10,000 data points per simulation were generated), statistical significance is not that surprising or particularly meaningful. Instead, the magnitude of effect sizes were assessed using Cohen’s^37^ rule of thumb as a guide (small effect = 0.01, medium effect = 0.059, large effect = 0.138). The statistic, eta squared (η^2^; Table 1), respectively show a major effect of a specific lipocalin on the distance from its active site and in ligand binding energy, a small effect between ligand distances and lipocalin binding energy, and a medium to large effect of the interaction between lipocalin and ligand binding energy and distance (Table 1). The relative effect sizes on the dependent variables (energy and distance) among the examined lipocalins and ligands can be seen in Fig. 5A.

The distance from the active site is relatively small for the respective control ligands of Japanin (cholesterol), Ra-HBP2 (histamine) and Monotonin (serotonin). The BBP, as a negative control, has all three ligands exploring at a greater distance from the active site. The Ra-HBP2 seems to also maintain serotonin close to the active site. Paesen *et al*.^38^ tested several amine compounds (including serotonin) to compete for Ra-HBP2-histamine binding, but no significant competition was observed among these related compounds. This, however, does not rule out the ability of Ra-HBP2 to sequester serotonin in an isolated system (i.e., not competing with histamine). For example, Monotonin also binds histamine at the L site, but has a 1.5 fold increase (more positive) change in enthalpy (*ΔH*) than Monomine^29^. Of the three *I. ricinus* salivary lipocalins, JAA67401 maintains serotonin and JAA70189 maintains histamine (at the H site) close to their respective active sites, similar to their respective positive controls. The lipocalin JAA65433, however, does not maintain any of the three ligands close to the respective active site (Fig. 5A).

In some systems, PELE overestimates the energy, specifically as the ligand escapes the binding cavity towards the solvent, e.g., JAA65433-serotonin. The best manner to relate PELE binding energy is through experimental *ΔH* measurements, since PELE implements a standard force field with calculations similar to *ΔH* (see Material and Methods). To demonstrate this relation and to further explore histamine at the L site, PELE simulations were started using the previous controls with Monomine replacing Ra-HBP2. The scatter plots in Fig. 5B shows that both Monomine and Montonin form a dense cluster of histamine exploration ~1 Å from the L site. The binding energy of Monotonin clusters at a higher range (−10 kcal/mol thru −25 kcal/mol) than Monomine (−15 kcal/mol thru −30 kcal/mol). Although Monotonin can bind histamine, it is (energetically) less favorable than serotonin^29^. The binding energy effect size of Monotonin-serotonin (−25 kcal/mol; Fig. 5A) is within the binding energy range of Monomine (Fig. 5B), which concurs with Mans *et al*.^29^ observations that both Monomine and Montonin have similar *ΔH* with their respective ligands. As negative controls for histamine binding, the crystal structures of Japanin and BBP form dense clusters 4 Å–6 Å away from the L site.

For the experimental *I. ricinus* lipocalins, JAA65433 falls within the range of Japanin and BBP (4 Å–6 Å), further indicating its inefficiency to maintain monoamines within the binding cavity, while JAA67401 clusters ~2 Å (Fig. 5B). The *I. ricinus* lipocalin JAA70189 forms a dense cluster of histamine exploration <1 Å from the L site. The NJ tree in Fig. 3 shows that JAA70189 clusters with Ra-HBP2 and given the positive results for H and L site histamine exploration suggests that JAA70189 may sequester two histamine molecules. Beaufays *et al*.^31^ discovered several *I. ricinus* lipocalins and, using binding assays, examined several ligands involved in inflammatory responses, including monoamines and leukotrienes. None of the *I. ricinus* lipocalins from the Beaufays *et al*.^31^ study bind monoamines and only one (LIR6 or Ir-LBP) binds LTB4^22^. The Ir-LBP can therefore act as a *bona fide* negative control for the simulations and modelling for monoamine binding. The Robetta server used Ra-HBP2 to model Ir-LBP, revealing a 1.9 Å backbone RMSD between the modelled Ir-LBP and the three *I. ricinus* lipocalins. The omega-loop is also slightly similar to JAA70189 and Ra-HBP2, unlike JAA67401 and JAA65433. The Ir-LBP-histamine exploration shows that the distance from the L site is similar to JAA67401 (~2 Å) with similar binding energy (Fig. 5B) suggesting that JAA67401 may only sequester serotonin. An additional Ir-LBP-serotonin exploration was executed to assure that JAA67401 maintains serotonin similar to Monotonin. The last scatter plot in Fig. 5B shows that the JAA67401-serotonin exploration forms a dense cluster ~1 Å from the L site, similar to Monotonin, while Ir-LBP explores between 2 Å–4 Å.

### Altered monoamine-binding modes by *I. ricinus* salivary lipocalins

The experimentally determined monoamine-binding residues in Fig. 4 are based on the Monotonin and Ra-HBP2 crystal structures. Crystal structures represent a static snapshot of, for instance, a protein-ligand complex. Protein-ligand interactions are not naturally static and, through its stochastic algorithm, PELE can explore several unreported interactions between a ligand and residues localized within the respective active site(s). The histograms in Supplemental Figure S3 show the residue-ligand contacts within 4 Å (pi-pi stacking are reported at 3.8 Å^39^) for the PELE explorations of serotonin (Monotonin and JAA67401) and histamine (Ra-HBP2 and JAA70189). The light grey histograms for Monotonin and Ra-HBP2 represent previously unreported residues that may form hydrophobic contacts or pi-pi stacking with their respective monoamine. For Monotonin these consist of four Val residues (Val43/45/75/84), Pro46, Ile82 and Met108. There are five previously unreported residues in Ra-HBP2 (Leu21, Phe67, Ile122, Thr109 and Val124).

**Figure 6:**
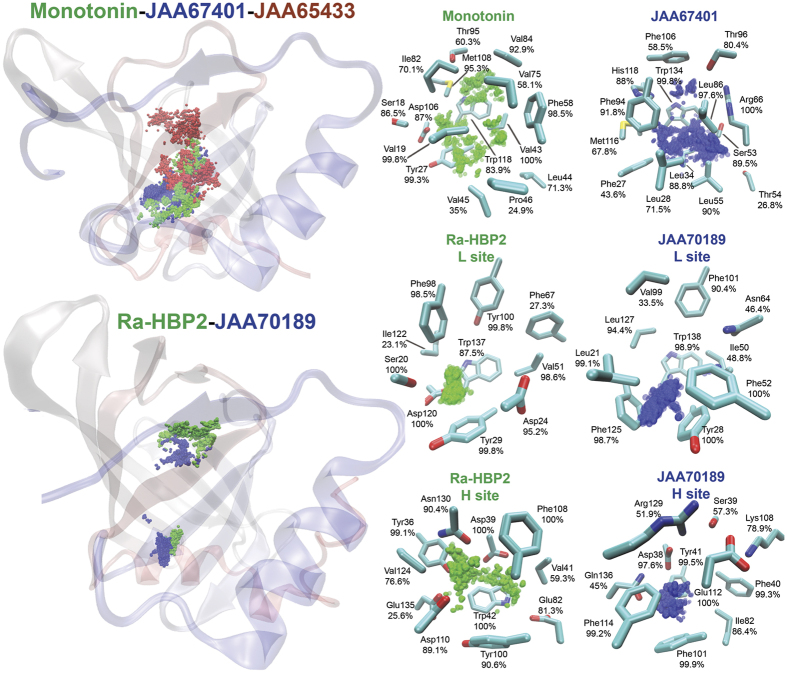
Binding modes of monoamine-binding lipocalins. The protein tertiary structures show the amine terminus position (balls) of the monoamines throughout the PELE simulations. The protein structures are color-coded form the N-terminus (red) to the C-terminus (blue). The top poses are the serotonin exploration for Monotonin (green), JAA67401 (blue) and JAA65433 (red), and the bottom shows the double histamine exploration for Ra-HBP2 (green) and JAA70189 (blue). To the right are localized representations for the monoamine explorations. Both Ra-HBP2 and JAA70189 depict both histamine-binding sites (H and L). The residues are labeled and the percentages represent those from Supplemental Figure S3 (>20%). The residues are atom color-coded (carbon = cyan; nitrogen = blue; oxygen = red; sulfur = yellow); hydrogen atoms are not shown.

The structural representations in Fig. 6 are of the monoamine explorations that show the interacting residues throughout the PELE simulations. For representation purposes, only residues that formed contact with the ligand for >20% during the PELE simulations are shown (according to those in Supplemental Fig. S3). Within the binding cavity, the major structural difference between the JAA67401 and JAA65433 orientation of Arg66 (Fig. 4) may influence their ability to maintain serotonin toward the closed-end of the beta-barrel. This containment is clearly seen by tertiary representation (Fig. 6) showing the migration of serotonin (only the amine is depicted as balls) of Monotonin (green) and JAA67401 (blue), and the escape of serotonin from the binding site in JAA65433 (red). Visual inspection of 70 lipocalins in the PDB showed that both these residue orientations are a structural commonality and its structural position alternates between beta-sheets C and G. Such extreme orientation and position of a residue close to the binding site seems to dictate the ability of JAA67401 and JAA65433 to maintain serotonin within the binding cavity. This residue and structural positions are also in concert with the main structural difference between Monomine and Monotonin, namely Tyr51 and Phe58, respectively^29^, which is homologous to His118Tyr in JAA67401 and JAA65433 shown in Fig. 4.

Both JAA67401 and JAA70189 have a more concentrated monoamine exploration within the binding cavity than Monotonin and Ra-HBP2, respectively (Fig. 6). The ligands for JAA67401 (serotonin) and JAA70189 (histamine) form contacts with the conserved Trp residue within the binding cavity at the closed-end (or the L site) that is involved in pi-pi stacking with monoamine aromatic rings^16^,^29^. The conserved Asp biomarker in both Monotonin (Asp101) and JAA67401 (Asp112) do not form contacts during the simulations. Although the *I. ricinus* lipocalins have substitutions for monoamine-binding residues compared to their respective templates (Fig. 4), Fig. 6 shows that these monoamine-binding residues are explored and at different structural positions (e.g., Ser53 and Thr96 in JAA67401).

### Expression profile of the three *I. ricinus* salivary lipocalins during tick life cycle and in response to pathogen infection

**Figure 7:**
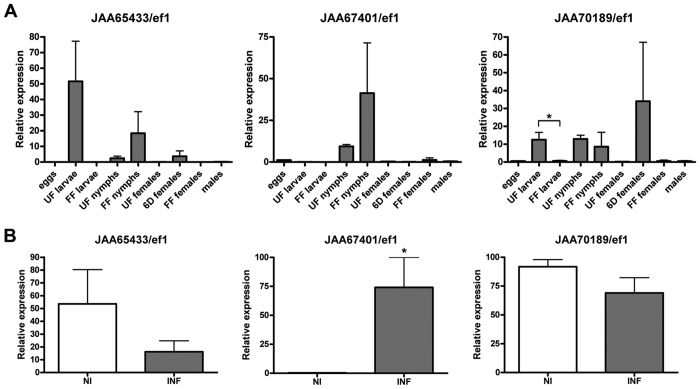
Expression and pathogenic infection profiles of the three *I. ricinus* salivary lipocalins. Panel A is the qPCR expression profiling of the three salivary lipocalins during blood feeding at different life cycle stages of *I. ricinus*. UF = unfed; 6d = fed for six days; FF = fully fed. Panel B is the relative mRNA expression for the three *I. ricinus* lipocalins in response to *B. afzelii* infection. NI = non-infected *I. ricinus* nymphs and INF = *B. afzelii* CB43 infected *I. ricinus* nymphs. The bars represent standard errors from three independent biological replicates. All qPCR results were normalized using the elongation factor 1 (ef1), a housekeeping gene. The asterisk (*) indicates significant differences (*P-value* < 0.05).

When assessed by qPCR, the expression patterns of the three *I. ricinus* salivary lipocalins differed between developmental stages and during feeding (Fig. 7A). JAA67401, a putative serotonin binder, was expressed predominantly in nymphs, whereas its isoform, JAA65433, was mainly expressed in unfed larvae, while expression of JAA70189, a putative histamine binder, appeared more widespread across stages and feeding. However, apart from the significant decrease in expression of JAA70189 during feeding of larvae (*P* = 0.044), differential expression of JAA65433, JAA67401 and JAA70189 genes during feeding at distinct life cycles was not significant because of the large variances (Fig. 7A). Nevertheless, the qPCR data (Fig. 7A) concur with the Illumina reads (Fig. 1) for these specific *I. ricinus* lipocalins. Both JAA65433 and JAA67401 display a similar pattern with higher Illumina reads in the late feeding stage (within 24 hours) during the nymphal life cycle with low reads during the adult life cycle (Fig. 1). The third lipocalin, JAA70189, has its own distinct pattern peaking in the early stages of feeding (within 48 hours) during the adult life cycle (Fig. 1). Of the three *I. ricinus* lipocalins, JAA65433 showed the least Illumina reads throughout (Fig. 1). The qPCR results, however, show that JAA65433 is highly expressed in unfed larvae compared to the other feeding and life cycles, and compared to the other two *I. ricinus* lipocalins (Fig. 7A).

The different expression profiles of the three *I. ricinus* lipocalins suggest different roles of the three lipocalins during feeding. Untalan *et al*.^40^ showed that the proteome of unfed *Rhipicephalus* (*Boophilus*) *microplus* larvae abundantly express a fatty acid binding protein although the functional significance is unknown. However, histamine and serotonin are known to have adverse effects on tick feeding though their roles vary with specific hosts. Cattle-feeding ticks (*R. appendiculatus*) secrete histamine-binding lipocalins but not serotonin-binding lipocalins whereas rodent–feeding ticks (*Dermacentor reticulatus*) secrete a lipocalin with two amine binding sites, one for histamine and the other for serotonin^41^. The three-host tick, *I. ricinus*, commonly feeds on rodents at the immature stage while preferring large mammals as adults; hence it has a need to control both serotonin (at the immature stage) and histamine (at the immature and adults stages).

The nymphal stage of *I. ricinus* is the most important developmental stage with regard to transmission of Lyme disease spirochetes to humans^42^. To investigate the expression of the three lipocalins upon pathogenic infection, *I. ricinus* nymphs were infected with *B. afzelii* CB43 strain and compared with non-infected nymphs. The putative serotonin-binder, JAA67401, showed significant upregulation (*P* = 0.031), suggesting a role in spirochete transmission; expression of JAA65433 and JAA70189 decreased upon *B. afzelii* infection but the change was not significant (Fig. 7B). Serotonin is particularly important in rodents in which it is produced and stored by tissue mast cells; in other mammals, serotonin is primarily secreted by blood platelets^43^. It may be in the interests of the Lyme disease spirochete to use its tick vector to control serotonin levels at the bite site.

## Concluding remarks

A new approach is presented here to predict the substrate(s) for highly divergent, uncharacterized proteins. Biophysical simulations, such as the stochastic approach employed by the PELE software, permit a robust analysis on the all-atom energy landscape exploration and interaction between a ligand and its target protein. The computational cost of testing high numbers of putative substrates (as is the case for lipocalins) renders PELE impractical. Therefore, we combined bioinformatics, phylogeny and structural analysis to reduce the number of putative candidates tested by PELE. By combining such methods with biophysics, disparate assumptions and calculations are used hence convergent results have a low probability of being by chance.

Accurate substrate prediction for uncharacterized proteins is a step forward during the high-throughput sequencing era. In our study, incorporating a multidisciplinary approach narrowed the scope of potential substrates for uncharacterized salivary lipocalins from the European vector of tick-borne pathogens, *I. ricinus*. From this multidisciplinary approach, we obtained highly significant and consistent results indicating that JAA67401 binds serotonin and JAA70189 binds two histamine molecules. How these lipocalins play a role in pathogen transmission will be further assessed since the serotonin-binding JAA67401 is upregulated upon *B. afzelii* infection.

## Materials and Methods

### Sequences

The protein sequence dataset for the global tree of lipocalins comprised 146 sequences. These lipocalins included 113 sequences analyzed by Ganfornina *et al*.^1^, 30 new sequences reported since 2000 with experimentally determined 3D structures, and 3 lipocalins described herein from the tick, *I. ricinus*. For the tick tree we used 1748 tick lipocalin sequences available in GenBank. From these initial sequences, those with less than 160 amino acids were removed since this is the average length of lipocalins, resulting in 1463 sequences. An alignment with such divergent sequences as that of lipocalins is challenging and no gap-free positions were obtained. Therefore in order to improve the alignment area, after the initial alignment using MAFFT, we removed from the alignment those sequences with many gaps using MaxAlign^44^. We did not use PAGAN (see below) at this stage, as it would be computationally expensive. The selection using MaxAlign resulted in an alignment of 788 sequences. Since MaxAlign excluded tick lipocalins with known ligands, we used the “add” option from the MAFFT package^45^ to include them. The final tick lipocalins alignment contained 803 sequences. The accession numbers of these sequences are provided in Supplemental File S1.

### Alignment optimization and phylogenetic analyses

A reliable Multiple Sequence Alignment (MSA) is a prerequisite for phylogenetic analysis. To test the reliability of the lipocalin family alignment, GUIDANCE was used, a tool for assigning a confidence score (from 0 to 1; 1 being best) for each residue, column, and sequence in an alignment^46^. The lipocalin amino acid sequences were aligned using the algorithms MAFFT^47^, PAGAN^34^ and CLUSTALW^48^, as implemented in GUIDANCE with 100 bootstrap repeats^46^. The Shapiro-Wilk normality test was used to test for normal distribution obtained from the GUIDANCE scores; normal distribution was rejected (*P-value* < 0.0001). A paired Wilcoxon test (of GUIDANCE sequence scores) and unpaired Mann-Whitney U test (GUIDANCE column scores) were used to test whether differences between scores were significant.

### Global tree of lipocalin amino acid sequences

The three alignments produced by the methods described in the previous section were used to infer maximum likelihood (ML), neighbor joining (NJ) and maximum parsimony (MP) phylogenetic trees. The best-fit model of sequence evolution was selected based on the Corrected Akaike Information Criterion (AICc) and the Bayesian Information Criterion (BIC) as implemented in Molecular Evolutionary Genetics Analysis software MEGA 6^49^. Accordingly, the WAG+G^50^ substitution model was chosen for subsequent phylogenetic analyses. The ML, MP and NJ methods implemented in MEGA 6^49^, were used to obtain the best tree topologies from each method. All sites of the alignments were used in the tree. The proportion of Gamma distributed sites was estimated in MEGA 6^49^. Reliability for internal branches was assessed using the bootstrapping method (1000 bootstrap replicates) implemented in MEGA 6^49^. Graphical representation and editing of the phylogenetic tree was performed with MEGA 6^49^.

### Tree of tick lipocalin amino acid sequences

The final alignment containing 803 was used to infer a NJ phylogenetic tree using a matrix of pairwise distances estimated under the Jones–Thornton–Taylor (JTT)^51^ model for amino acid sequences implemented in MEGA 6^49^. Reliability for internal branches, graphical representation and editing of the phylogenetic tree was performed as explained above. The resulting tree is available in newick format in Supplemental File S1.

### Tertiary structures of lipocalins

The crystal structures for the biophysical simulations (see section below) were downloaded from the PDB. These PDBs included Japanin (PDB: 4BOE) and Ra-HBP2 (PDB: 1QFT) from *R. appendiculatus*, Monotonin from the soft tick *A. monolakensis* (PDB: 3BRN), and the bilin-binding protein (BBP; PDB: 1BBP) from the butterfly *P. brassicae*. The three *I. ricinus* salivary lipocalins were modelled using the Robetta server^52^. All structures were optimized using the Schrödinger’s Maestro Protein Preparation Wizard^53^ and any steric clashes identified were removed via minimization - default conditions in the Schrodinger’s Maestro package. The Protein Preparation Wizard analyzes the input structure for clustering hydrogen bonds with sampling at the highest degree of hydrogen bonding in equilibrium. A total of 100000 Monte Carlo orientations are performed for each cluster. The algorithm determines an optimized structure according to electrostatic and geometric scoring functions.

### Metropolis Monte Carlo exploration simulations for ligand screening

The Metropolis Monte Carlo-based PELE server was used for the biophysical simulations. The PELE software can demonstrate, step-by-step, the entrance or exit pathway of a ligand to/from the active site of a protein. Since lipocalins maintain ligands within their eight-stranded beta-barrel, the PELE software can demonstrate the efficiency by which a particular lipocalin can maintain a specific ligand within the binding cavity. The PELE server provides ready-made scripts that can be accessed at https://pele.bsc.es/ and its many applications are thoroughly explained elsewhere^24^. The PELE software implements an anisotropic network model (ANM)^54^ for perturbations of the alpha-carbon backbone causing structural conformational changes. Molecular dynamics studies on lipocalins indicate that the overall conformation of the beta-barrel is maintained while larger changes occur for the open-end beta-hairpin loops^55^. Confirmed by NMR, these large conformational changes are present for open-end beta-hairpin loops in bound and unbound lipocalin structures^56^. Some regions were omitted from ANM calculations to minimize extraneous perturbations outside the open-end beta-hairpin loops. There are examples of Normal Mode Exploration at the PELE website (https://pele.bsc.es/) for more details.

Briefly, the PELE algorithm performs three stages. First, localized ligand translations (or perturbations) and alpha-carbon protein backbone perturbations are performed. Small translations (tra_r 0.6) were used for a more local exploration (see binding refinement script at the PELE website (https://pele.bsc.es/). Secondly, side chains are optimized with steric filters with a rotamer library^57^. At the third stage, PELE performs a minimization with a truncated Newton minimizer within a surface generalized Born implicit solvent^58^ to reach a local minimum. These three stages are iterated in parallel with several computer-processing units resulting in a series of frames. The PELE algorithm implements a Metropolis Monte Carlo criterion that either accepts a step if it is equal to and/or less than the initial energy; it rejects the step if it is greater than the initial energy. The PELE energy of the entire system is calculated by a standard force field known as the optimized potentials for liquid simulations (OPLS-2005)^59^. The OPLS calculations are correlated with enthalpy of vaporization experiments^60^. The binding energy (kcal/mol) equals the lipocalin-ligand complex minus the sum of its individual units for the lipocalin and ligand. Images and ligand-residue contacts were performed using the Visual Molecular Dynamics platform^61^.

### Statistical analysis of ligand exploration simulations

A two-way ANOVA was performed to test the differences in the response variable of the distance (in Å) from the active site and binding energy (in kcal/mol), as calculated by PELE, and for the categorical explanatory variables ‘lipocalin’ and ‘ligand’. An ANOVA, which assumes the data are normally distributed, was chosen as the appropriate statistical test because i) it is robust to deviations from normality, even in small sample sizes, and ii) in very large sample sizes, as in our data, the central limit theorem (CLT) validates normal-based methods, such as ANOVA^62^,^63^. However, frequency distribution histograms were also examined to confirm that there were no radical departures from normality. The tests and plots were performed using the R statistical package^64^.

### Relative quantitative RT-PCR analyses

Total RNA was isolated from eggs, larvae, nymphs (non-infected and infected with *B. afzelii* CB43) and adult tick homogenates as well as from dissected tissues (salivary glands, guts and ovaries) from semi-engorged *I. ricinus* females, as previously described^65^. For the qPCR, the single-stranded cDNA that was reverse-transcribed using 0.5 μg of total RNA served as a template for an expression profile analyses using the LightCycler 480 (Roche) and SYBR green chemistry. The cDNA preparations from developmental stages, *B. afzelii* CB43 infection and dissected tissues were made in independent biological triplicates. The qPCR statistics was analyzed and the histograms were generated using GraphPad Prism 6 for Windows (GraphPad Software, San Diego, USA). An unpaired Student’s t-test was used for sample group comparisons and for evaluating statistical significance (*P-value* < 0.05). The error bars show standard error.

For the *B. afzelii* CB43 infection, the infestation technique, time points and detection methods were identical to those performed by Golovchenko *et al*.^66^. Briefly, cultured *B. afzelii* CB43 were used to infect C3H/HeN mice (Jackson Laboratory, Sulzfeld, Germany) via subcutaneous injection. On the 4^th^ week after inoculation, pathogen-free, laboratory reared, *I. ricinus* larvae (~100/mouse) were fed on infected mice and then left to molt to nymphs. Successful infection of *B. afzelii* CB43 was determined if >80% of nymphs were infected, as confirmed via PCR. Uninfected nymphs (i.e., the control group) were treated the same, but larvae were fed on uninfected mice. All laboratory animals were treated in accordance with the Animal Protection Law of the Czech Republic No. 246/1992 Sb., ethics approval No. 137/2008. All animal experiments were conducted following guidelines from the Czech Ministry of Agriculture with protocols approved by the Institute of Parasitology Animal Care and Use Committee.

## Additional Information

**How to cite this article**: Valdés, J. J. *et al*. Substrate prediction of *Ixodes ricinus* salivary lipocalins differentially expressed during *Borrelia afzelii* infection. *Sci. Rep.* **6**, 32372; doi: 10.1038/srep32372 (2016).

## Supplementary Material

Supplementary Information

## Figures and Tables

**Table 1:** Descriptive statistics for two-way ANOVA on the distance from orthosteric (active) binding site.

Source of Variation	Degrees of freedom	Sum of squares	Mean squares	*F*-value	*P-value*	*Eta squared*
Distance from respective active binding site						
Lipocalin	6	1484160	247360	130705	<2e-16***	0.35
Ligand	2	118875	59437	31407	<2e-16***	0.03
Lipocalin*Ligand	12	735220	61268	32374	<2e-16***	0.18
Error	981602	1857683	2			0.44
Binding Energy						
Lipocalin	6	5060448	843408	36146	<2e-16***	0.05
Ligand	2	67148237	33574119	1438876	<2e-16***	0.63
Lipocalin*Ligand	12	10717961	893163	38278	<2e-16***	0.10
Error	981602	22904288	23			0.22

^***^Highly significant effect.
