# Supplementary material for: Substrate prediction of Ixodes ricinus salivary lipocalins differentially expressed during Borrelia afzelii infection
Source: Sci Rep. 2016 Sep 1;6:32372. doi: 10.1038/srep32372 (PMC5008119; doi:10.1038/srep32372)
Supplement: Supplementary Information [file srep32372-s1.pdf]

Substrate prediction of *Ixodes ricinus* salivary lipocalins differentially expressed during *Borrelia afzelii* infection

James J. Valdés[1,2*†], Alejandro Cabezas-Cruz[3†], Radek Sima[1], Philip T. Butterill[4], Daniel Růžek[1,2,4‡] and Patricia A. Nuttall[5‡]

[1]*Institute of Parasitology, The Czech Academy of Sciences, Branišovská 31, CZ-37005 České Budějovice, Czech Republic*

[2]*Department of Virology, Veterinary Research Institute, Hudcova 70, CZ-62100 Brno, Czech Republic*

[3]*Center for Infection and Immunity of Lille (CIIL), INSERM U1019 – CNRS UMR 8204, Université Lille Nord de France, Institut Pasteur de Lille, Lille, France*

[4]*Biology Center, The Czech Academy of Sciences, University of South Bohemia, Branišovská 31, CZ-37005 České Budějovice, Czech Republic*

[5]*Department of Zoology, University of Oxford, Oxford, OX1 3PS, UK*

[†]Joint first authorship; [‡]Joint senior authorship

***Corresponding Author:**

James J. Valdés, Ph.D.

E-mail: valdjj@gmail.com

Tel: +420387775491

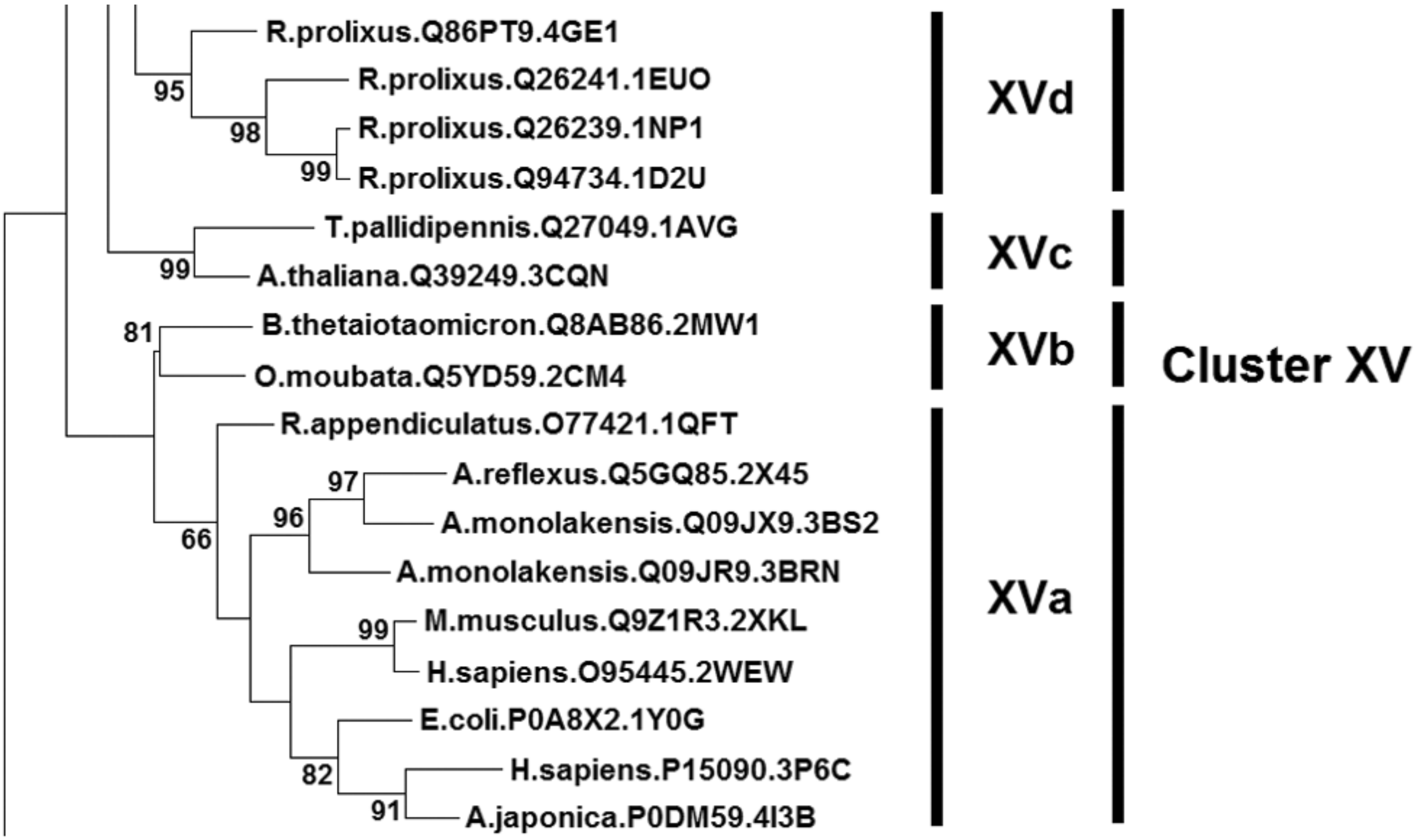

Supplemental Figure S1. Phylogenetic organization of Cluster XV. The subclusters formed by Cluster XV as shown in Figure 1 are depicted in detail.

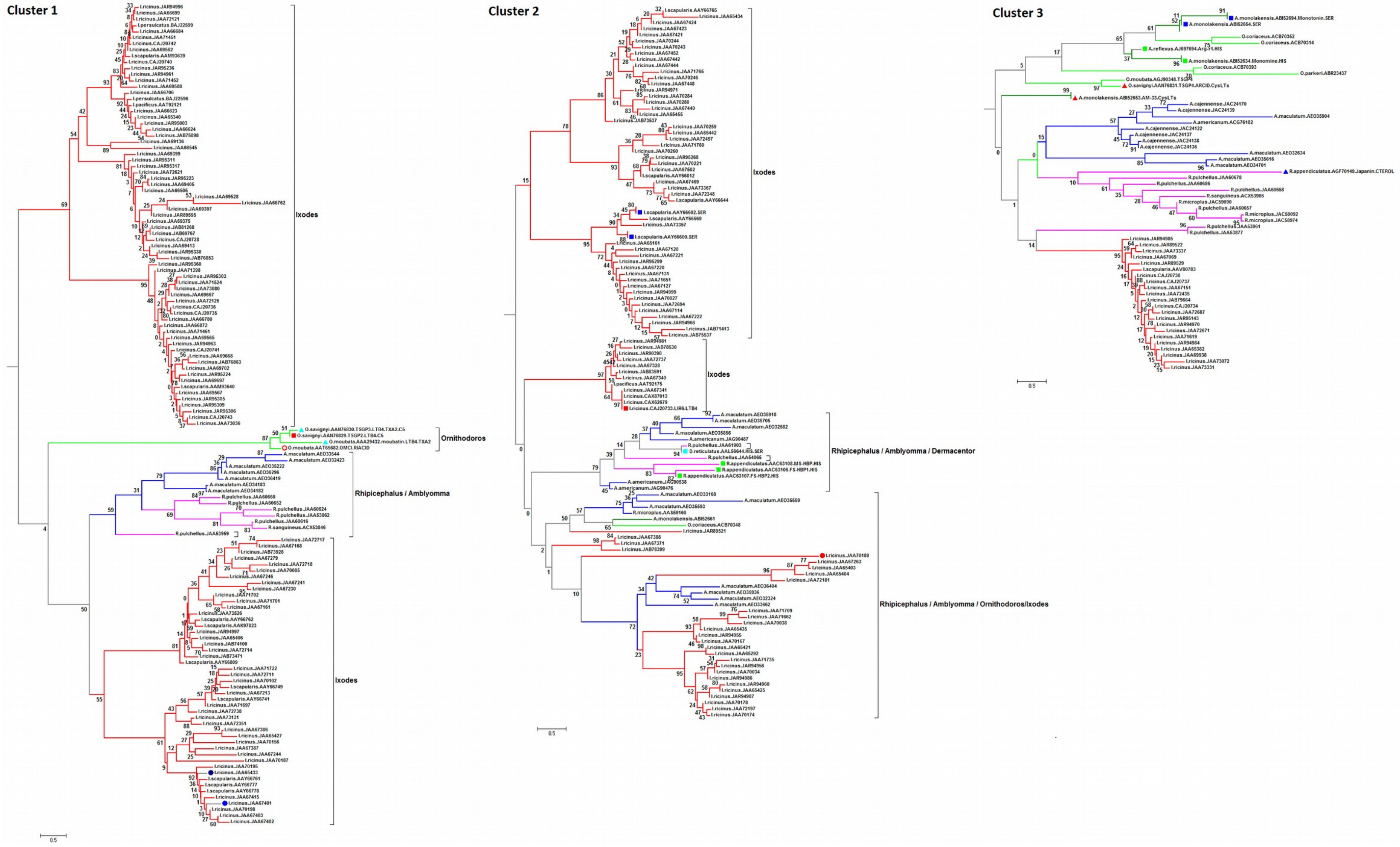

Supplementary Figure S2. Phylogenetic tree of tick lipocalins. Different tick genera are shown (colored branches). Tick lipocalins known substrate (geometric colored symbols) were included in the analysis. Abbreviation for substrate are as follow: ricinoleic acid (RIACID), leukotrienes B4 (LTB4), thromboxane A2 (TXA2), histamine (HIS), serotonine (SER), cholesterol (CTEROL) and cysteinyl leukotrienes (CYSLTS).

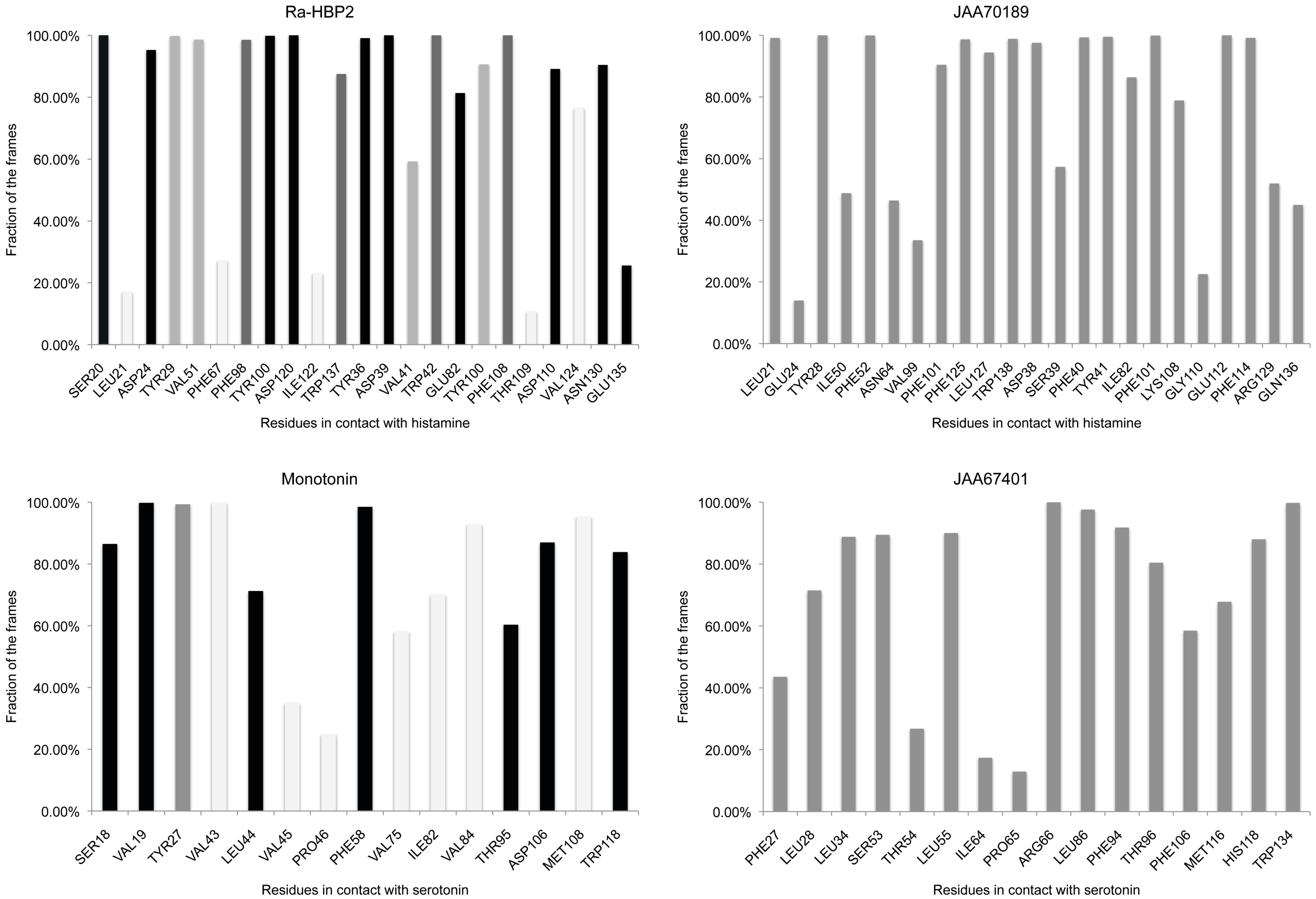

Supplementary Figure S3. Residue contact histograms. The histograms represent the fraction of the frames during the PELE simulations (y-axis) of residues that form contact with the ligand (x-axis). The different shaded histograms for the control crystal structures Monotonin and Ra-HBP2 depict the experimentally determined residues that form contact with their respective ligands. Black = contact; dark grey = pi-pi stacking; grey = hydrophobic contacts; light grey = previously unreported.

Supplemental Table S1. *Ixodes ricinus* lipocalins - primers for qPCR

Accession # (Protein/mRNA)	Primer	Sequence 5'→3'	Product size
JAA70260_GADI01003548	Forward	ACCGATCCCTCGACTACCTT	150 bp
	Reverse	GACACGGCAGCTTTGGTTAT	
JAA67230_GADI01006578	Forward	TAAAAGCCCGCTGAGACACT	141 bp
	Reverse	GGTTGGTTGTTTCCTGCACT	
JAA67401_GADI01006407	Forward	GGACAAATGGGATGTGCTCT	106 bp
	Reverse	CAACTTGATGGCTTGTTTCG	
JAA70156_GADI01003652	Forward	AGTGATGGCCGAATAGGATG	117 bp
	Reverse	ATATACGTGTCGGGGGTTCA	
JAA65433_GADI01008375	Forward	CCCGCTCCTCTACTGTGAAC	89 bp
	Reverse	CAAGGTCATCACAGGCTTCTT	
JAA70280_GADI01003528	Forward	ACAAGACATTGCAGCCTGTG	120 bp
	Reverse	TCTTGCGGGATCATCGTTAT	
JAA70189_GADI01003619	Forward	TTGCGGCTCTTCTAACCAGT	131 bp
	Reverse	GGAACGATACAGCAGGTGGT	
JAA65455_GADI01008353	Forward	GTGGGTCAACTGTCGGCTAT	124 bp
	Reverse	ACCGTGTTTGTGTCCCAAGT	

Supplemental Table S2. Expression profiling of eight *I. ricinus* lipocalins in semi-engorged adult female *I. ricinus*

	Gene expression		
**Accession # (Protein/mRNA)**	**SG**	**GUT**	**OV**
JAA70260_GADI01003548	NO	NO	NO
JAA67230_GADI01006578	NO	NO	NO
**JAA65433_GADI01008375**	YES	YES*	YES*
JAA70156_GADI01003652	NO	NO	NO
**JAA67401_GADI01006407**	YES	NO	NO
JAA70280_GADI01003528	NO	NO	NO
**JAA70189_GADI01003619**	YES	NO	NO
JAA65455_GADI01008353	NO	NO	NO

The sequences were originally determined by Schwarz et al. (2013).

SG = salivary glands, GUT = midgut, OV = ovaries, *weak expression

Bold, underlined accession numbers represent expressed genes.

Supplemental Table S3. Reliability of phylogenetic analyses using different alignment algorithms

**Phylogenetic method**	**Neighbor joining**			**Maximum Parsimony**			**Maximum Likelihood**		
**Alignment algorithms**	**PAGAN**	**MAFFT**	**CLUSTALW**	**PAGAN**	**MAFFT**	**CLUSTALW**	**PAGAN**	**MAFFT**	**CLUSTALW**
**Clusters**	**Bootstrap values/status of the cluster**			**Bootstrap values/status of the cluster**			**Bootstrap values/status of the cluster**		
Cluster I	94	99	99	83	62	94	97	99	98
Cluster II	Expanded*	35	Expanded*	Fragmented**	Fragmented**	37	Expanded*	Expanded*	Fragmented**
Cluster III	93	99	99	61	98	99	80	99	96
Cluster IV	96	98	99	95	86	87	98	80	91
Cluster V	30	14	22	38	Fragmented**	Fragmented**	30	12	66
Cluster VI	99	99	99	98	86	92	99	97	88
Cluster VII	99	99	99	93	98	99	96	73	99
Cluster VIII	85	90	89	82	97	97	96	94	94
Cluster IX	99	99	99	99	98	99	99	99	99
Cluster X	59	97	97	30	12	87	86	92	94
Cluster XI	98	Fragmented**	Fragmented**	96	40	Fragmented**	97	67	72
Cluster XII	99	99	99	98	98	99	99	98	94
Cluster XIII	92	79	99	68	75	97	99	83	95
Cluster XIV	94	98	93	84	84	87	95	92	92
Cluster XVa	30	Fragmented**	Fragmented**	58	Fragmented**	Fragmented**	66	11	Fragmented**
Cluster XVb	52	Fragmented**	Fragmented**	79	Fragmented**	Fragmented**	81	5	Fragmented**
Cluster XVc	98	99	Fragmented**	90	96	Fragmented**	99	96	Fragmented**
Cluster XVd	82	99	Fragmented**	68	96	Fragmented**	95	99	Fragmented**

*Including Cluster I

**Distributed in different Clusters
